# Predicting Outcomes to Optimize Disease Management in Inflammatory Bowel Diseases

**DOI:** 10.1093/ecco-jcc/jjw116

**Published:** 2016-06-09

**Authors:** Joana Torres, Flavio Caprioli, Konstantinos H. Katsanos, Triana Lobatón, Dejan Micic, Marco Zerôncio, Gert Van Assche, James C. Lee, James O. Lindsay, David T. Rubin, Remo Panaccione, Jean-Frédéric Colombel

**Affiliations:** ^a^Surgical Department, Gastroenterology Division, Hospital Beatriz Ângelo, Loures, Portugal; ^b^Department of Pathophysiology and Transplantation, Università degli Studi di Milano and Gastroenterology and Endoscopy Unit, Fondazione IRCCS Cà Granda, Ospedale Policlinico di Milano, Milan, Italy; ^c^Division of Gastroenterology, Department of Internal Medicine, School of Medical Sciences, University of Ioannina, Ioannina, Greece; ^d^Department of Gastroenterology, Germans Trias i Pujol University Hospital, Badalona, Barcelona, Spain; ^e^University of Chicago Medicine Inflammatory Bowel Disease Center, Chicago, IL, USA; ^f^Inflammatory Bowel Disease Unit, Potiguar University School of Medicine, Natal, Brazil; ^g^Division of Gastroenterology and Hepatology, University Hospitals Leuven, Leuven, Belgium; ^h^Department of Medicine, University of Cambridge, Cambridge, UK; ^i^Barts Health NHS Trust, Royal London Hospital, Whitechapel, London, UK; ^j^Inflammatory Bowel Disease Clinic, Division of Gastroenterology and Hepatology, University of Calgary, Calgary, Alberta, Canada; ^k^The Henry D. Janowitz Division of Gastroenterology, Icahn School of Medicine at Mount Sinai, New York, NY, USA

**Keywords:** Complications, Crohn’s disease, disease progression, prognostic factors, risk factors, ulcerative colitis

## Abstract

**Background and Aims:**

Efforts to slow or prevent the progressive course of inflammatory bowel diseases [IBD] include early and intensive monitoring and treatment of patients at higher risk for complications. It is therefore essential to identify high-risk patients – both at diagnosis and throughout disease course.

**Methods:**

As a part of an IBD Ahead initiative, we conducted a comprehensive literature review to identify predictors of long-term IBD prognosis and generate draft expert summary statements. Statements were refined at national meetings of IBD experts in 32 countries and were finalized at an international meeting in November 2014.

**Results:**

Patients with Crohn’s disease presenting at a young age or with extensive anatomical involvement, deep ulcerations, ileal/ileocolonic involvement, perianal and/or severe rectal disease or penetrating/stenosing behaviour should be regarded as high risk for complications. Patients with ulcerative colitis presenting at young age, with extensive colitis and frequent flare-ups needing steroids or hospitalization present increased risk for colectomy or future hospitalization. Smoking status, concurrent primary sclerosing cholangitis and concurrent infections may impact the course of disease. Current genetic and serological markers lack accuracy for clinical use.

**Conclusions:**

Simple demographic and clinical features can guide the clinician in identifying patients at higher risk for disease complications at diagnosis and throughout disease course. However, many of these risk factors have been identified retrospectively and lack validation. Appropriately powered prospective studies are required to inform algorithms that can truly predict the risk for disease progression in the individual patient.

## 1. Introduction

Crohn’s disease [CD] and ulcerative colitis [UC] are chronic gastrointestinal inflammatory diseases characterized by disabling bowel symptoms. Ongoing inflammation leads to progressive bowel damage and complications, often requiring surgery. These diseases are associated with significant morbidity, resource utilization and costs to society. Furthermore, as these inflammatory bowel diseases [IBD] often present early in life, they can compromise education, career development and family planning.

The recognition that chronic uncontrolled inflammation in IBD ultimately results in poor outcomes has led to a recent paradigm shift in treatment, with the belief that early intervention with immunosuppressant and biological therapy can prevent disease progression and avoid complications.^1,2,3,4,5^ However, treatment of all patients with biologics and/or combination therapy is economically unsustainable and would risk exposing those with an indolent disease course to unnecessary risks or side effects of potent therapy. The challenge remains to select the patients who will benefit most from early intensive therapy, while sparing those who will derive minimal benefit from such treatment.^6^ The ability to predict specific disease complications such as progression of phenotype from inflammatory to penetrating or fibrostenotic disease, need for surgery or development of dysplasia or cancer would be of particular value, as would being able to identify ‘red flags’ that could alert the clinician to an impending flare or relapse.

As part of the IBD Ahead 2014 educational programme, we conducted a comprehensive literature review to identify predictors of long-term IBD prognosis and generate draft expert summary statements relating to prognosis. Statements were refined at national meetings of IBD experts and were finalized at an international meeting in November 2014. Here we present the agreed statements, together with a summary of published evidence, to guide clinicians on the best use of these predictors in the individual patient.

## 2. Methods

In February 2014, the Global Steering Committee [GSC] of the IBD Ahead 2014 educational programme identified key topics of interest or uncertainty in understanding prognostic factors in IBD and developed clinical questions relating to these topics. Six bibliographical fellows [FC, KK, TL, DM, JT and MZ] were nominated by the GSC to identify and evaluate the published evidence on prognostic factors under the mentorship of JOL, GVA, DR, J-FC and JCL, respectively. PubMed, Embase and the Cochrane Library were searched using pre-defined search strings and limits, and additional searches were conducted by hand as required. Searches were restricted to manuscripts published in English after 1993. Abstracts from the following conferences were also searched: European Crohn’s and Colitis Organisation Congress 2013, 2014; Digestive Disease Week 2013, 2014; and United European Gastroenterology Week 2012, 2013. The bibliographical fellows reviewed the evidence and developed summary statements in response to the clinical questions, with evidence levels [ELs] assigned to each statement based on the University of Oxford Centre for Evidence-Based Medicine 2011 criteria [http://www.cebm.net/index.aspx?o=5653]. In June and July 2014, the literature review subgroup [JOL, JCL, GVA] and the programme co-chairs [J-FC, RP] reviewed and simplified the statements. National meetings were held in August–November 2014 in the 32 participating countries to allow participants to review, vote and provide their expert opinions and local perspective on the statements. Based on this feedback, a set of consolidated statements was generated by the GSC focusing on predictors of long-term IBD prognosis. An international meeting was held in November 2014 with 99 experts from each of the participating countries. Participants voted on their level of agreement with each statement using a scale of 1 to 9 [where 1=strong disagreement and 9=strong agreement]. If ≥75% of participants scored within the 7–9 range, then the statement was deemed to be agreed upon. If <75% of participants scored within this range, the statement was debated and revised, and a second vote was taken. Again, if ≥75% of participants scored within the 7–9 range, the statement was deemed to be agreed upon. If agreement was not reached at this stage, a lack of agreement was noted. The agreed statements and a summary of the supporting evidence are presented here.

## 3. Results

### 3.1. Question 1: What are the prognostic factors for disease progression – change in disease behaviour [B1 to B2 and B3], need for therapy escalation, perianal disease, bowel damage and disability – in CD?

**Table:** 

**Summary statements**		**Agreement [score 7–9], *n*/*N* [%]**
1	Ileal disease location [EL2], upper gastrointestinal [GI] involvement [EL3] and extraintestinal manifestations [EIMs] [EL3] are associated with disease progression to complicated behaviour* in CD.	66/80 [82%]
2	Younger age and perianal disease at diagnosis are associated with a disabling course of CD [EL3].	76/83 [91%]
3	Smoking predicts increased need for therapy escalation [EL3], progression to complicated disease behaviour [EL3], need for surgery [EL3] and post-operative recurrence in CD [EL3].	80/92 [87%]
4	Endoscopic severity of CD may be associated with development of penetrating complications [EL4].	73/93 [79%]
5	Serological reactivity to certain microbial antigens is associated with progression to complicated disease behaviour in paediatric and adult-onset CD [EL2]; the risk of disease evolution towards complicated forms of CD increases with the number of antibodies detected in the serum [EL2].	72/91 [79%]
6	Although mutations in some genes (such as NOD2 [EL2]) may be associated with progression to complicated CD, as yet there is no evidence for use of genetic markers in clinical practice.	92/98 [94%]

*B2/B3 behaviour.

CD is an inflammatory disease that may affect the entire GI tract, with repeated flares resulting in bowel damage that leads to a complicated disease course and short- and long-term disability. Therapy may involve repeated courses of corticosteroids and ultimately surgery.^7^ Our literature search identified a number of studies evaluating risk factors for complicated CD outcomes [Table 1]; however, interpretation was hindered by the lack of consistent or standard definitions of disease progression. Interestingly, we did not find any studies documenting risk factors for bowel damage or participation-related disability.

**Table 1.** Clinical, demographic and endoscopic prognostic predictors in Crohn’s disease [CD] and the associated impact on the disease course.

**Prognostic factor**	**Impact**
Young age at diagnosis	• Disabling CD^a^ [< 40 years]^8 ^ • Need for surgery^8,11,24,38,51 ^ • More frequent L4 disease [paediatric patients]^119,120 ^ • More frequent extensive disease [paediatric patients] ^119,120 ^ • Intestinal failure^13 ^
Requirement for steroids at diagnosis	• Disabling CD^a^ ^8 ^
Complicated behaviour [B2 and/or B3]	• Surgery^9,38,42,48 ^ • Hospitalization^54,86 ^
Ileal disease[L1] and ileocolonic disease [L3]	• Surgery^28,38,45 ^ • Disabling CD^a^ ^8 ^ • Complicated behaviour^b^ ^16 ^ • Disease behaviour progression^16 ^ • Time to hospitalization^54,86 ^
Colonic CD	• Inflammatory phenotype^51 ^ • Milder course [protective from hospitalization and surgery]^22,38 ^ • Permanent stoma [distal disease, severe rectal disease, rectal resection]^57 ^
Upper GI extent [L4]	• Complicated behaviour^15 ^ • Hospitalization^81 ^ • Multiple surgeries^15 ^
Perianal disease	• Disabling CD*^8 ^ • Permanent stoma [refractory perianal disease, anal canal stricture, complex fistulizing disease]^57 ^
Deep ulcerations at index colonoscopy	• Surgery^26 ^ • Penetrating complications^26 ^
Smoking	• Complicated CD [disease progression]^75 ^ • Higher therapeutic requirements^20 ^ • Risk for first surgery [conflicting evidence]^22,48 ^
Positive antimicrobial markers	• Risk of complicated phenotype and surgery [increasing with higher number of positive antibodies and higher titres]^43 ^
*NOD2* mutations	• Ileal disease^37 ^ • Risk for surgery^33 ^

^a^As defined by Beaugerie.^8^

^b^B2 and/or B3.

Two large tertiary-centre studies provided the greatest insight into clinical predictors of disability and severe disease in CD.^8,9^ The primary outcome in these studies was disabling disease, defined as a requirement for more than two steroid courses, steroid dependence, hospitalization for disease flare or complication, disabling chronic symptoms for a cumulative time of more than 12 months, or need for immunosuppressive therapy, intestinal resection or surgery for perianal disease. Variables assessed included gender, ethnicity, age at onset, disease location, previous appendectomy, smoking status, EIMs, perianal lesions at diagnosis and the need for steroids for treating the first flare-up. In the first study, an initial requirement for steroids (odds ratio [OR] 3.1; 95% confidence interval [CI] 2.2–4.4), age at diagnosis of less than 40 years [OR 2.1; 95% CI 1.3–3.6] or the presence of perianal disease at diagnosis [OR 1.8; 95% CI 1.2–2.8] were independently associated with disabling disease.^8^ In the second study, independent predictors of disabling CD were an initial requirement for steroids [OR 1.7; 95% CI 1.0–2.7], perianal lesions at diagnosis [OR 2.6; 95% CI 1.4–5.1] and ileocolonic location of the disease [OR 1.7; 95% CI 1.1–2.8].^9^ Another study found that older age at diagnosis and the absence of perianal lesions predicts a milder CD course.^10^ One of the most important clinical risk factors for disease progression is disease location [ileal and ileocolonic vs colonic, rectal involvement, and upper GI involvement].^11–15^ In a population-based cohort from North America, disease in the terminal ileum, ileocolonic disease and upper GI involvement were strongly associated with change in disease behaviour in a multivariate model.^16^

Other factors that may act as predictors of disease progression include smoking, endoscopic disease severity and serological status. In several studies in CD patients, active smoking was associated with stenosing/fistulizing behaviour,^17,18,19^ the need for steroids or immunomodulators,^20^ requirement for surgery^21,22^ and disease recurrence following surgery.^23,24^ In one study, passive smoking was associated with increased need for immunosuppressants and biologics in CD patients.^25^

In the only study identified that specifically assessed the value of endoscopic findings in CD, severe endoscopic lesions [defined as extensive and deep ulcerations covering more than 10% of the mucosal area of at least one segment of the colon] at index colonoscopy were associated with penetrating complications in CD.^26^

While the prognostic value of inflammatory markers on CD course is limited,^27,28,29^ results from several independent adult and paediatric cohorts have indicated that circulating antibodies against bacterial antigens are associated with complicated CD and evolution towards stricturing or penetrating behaviour.^30,31^ Interestingly, a direct correlation was observed between the magnitude of immune response to microbial antigens and frequency of penetrating/stenosing disease in children. Additionally, paediatric CD patients who were positive for one or more immune response progressed to penetrating or stricturing disease sooner after diagnosis compared with those negative for all immune responses.^31^

An association between mutations in the *NOD2* gene and complicated disease phenotype has been suggested in several independent cohorts^32,33^ and a meta-analysis.^34^ However, the low effect size of other CD-associated genetic polymorphisms means that a very large sample size is required to make true associations between genotype and phenotype. Current studies have lacked power to establish a true association and do not adequately take into account the effects of age, disease location, smoking and other variables on phenotype. Moreover, several studies have reported that *NOD2* mutations are specifically associated with stricturing ileal disease, which almost certainly accounts for the reported association with increased rates of surgery.^35,36^ Indeed, this association disappears if the data are corrected for disease location.^37^

### 3.2. Question 2: What are the prognostic factors for surgery or multiple surgeries in CD?

**Table:** 

**Summary statements**		**Agreement [score 7–9], *n*/*N* [%]**
1	Younger age at diagnosis [adults <40 years] increases risk of surgery [EL2]; in paediatric patients, younger children have lower risk for surgical resection [EL3].	85/96 [89%]
2	Disease located in the small bowel carries a higher risk for surgery than isolated colonic disease [EL2].	86/90 [96%]
3	Penetrating and stricturing phenotypes at diagnosis are independent risk factors for surgery [EL2].	92/99 [93%]
4	Extensive and deep ulcers at colonoscopy in patients with colonic CD may predict the need for surgery [EL4].	79/88 [90%]
5	NOD2/CARD15 polymorphisms and/or anti-*Saccharomyces cerevisiae* antibodies [ASCA]-positive status may be associated with an increased risk of surgery [EL2].	72/82 [90%]

The cumulative probability of surgery in CD has been evaluated in several population-based cohorts.^21,38,39,40^ A number of clinical risk factors have been associated with an increased need for surgery [Table 1]. In adult patients, younger age at diagnosis is a prognostic factor for surgery.^38,39,41^ Within the paediatric population, available data suggest an opposite trend, however, with younger age at diagnosis associated with a decreased risk of surgery.^42,43,44^ One potential explanation for this observation is that younger paediatric patients present more commonly with isolated colonic disease,^42^ which has been repeatedly associated with lower surgical rates [see below].

Disease location is among the most important prognostic factors for surgery in CD, and should actively be incorporated into clinical decision-making. Disease located in the small bowel [ileal/ileocolonic disease] has been consistently identified as an independent risk factor for surgery in adult populations,^21,38,45,46,47,48^ possibly because small bowel disease is more frequently associated with penetrating and stenosing behaviour.^22^ Disease located in the jejunum and upper GI tract [L4] is also indicative of higher surgical risk for similar reasons,^11,12,13,14,15,49^ while colonic disease [L2] is protective against major surgery.^22,42,49,50,51^

Penetrating and stricturing disease at diagnosis is possibly the most important independent factor associated with the need for surgery.^21,38,42,48^ Furthermore, patients who have surgery for penetrating complications have a higher likelihood of being re-operated on and a shorter time to second surgery.^15,52^ Extensive and deep ulcerations on index colonoscopy have been shown in a retrospective study to represent an independent risk factor for surgery, in patients with colonic disease.^26^

A large systematic review and meta-analysis found that the risk of any CD surgery was increased by 58% if any *NOD2* mutation was present, with a pooled sensitivity of 41% and a specificity of 74%.^34^ A meta-analysis of seven cohort studies and four case-control studies showed an association between ASCA-positive status and surgery risk [OR 1.64; 95% CI 1.37–1.95].^53^ In addition, greater immune responses to anti-Cbir1, anti-ompC, ASCA and perinuclear anti-neutrophil cytoplasmic antibody [p-ANCA] were shown to be predictive of surgery in a paediatric population.^43^ The increased risk of surgery associated with these genetic and serological markers probably also reflects their association with ileal and ileocolonic disease and complicated disease behaviour.

### 3.3. Question 3: What are the prognostic factors for hospitalization in CD?

**Table:** 

**Summary statement**		**Agreement [score 7–9], *n*/*N* [%]**
1	Penetrating and stricturing phenotypes predict hospitalization and re-hospitalization [EL2].	68/74 [92%]

Hospitalization is generally regarded as a marker of high disease activity or severity in CD. However, our review of the literature revealed that very few studies have assessed predictive factors of hospitalization in patients with CD [Table 1]. Generally, risk factors associated with disease progression and risk for surgery are also associated with higher likelihood of hospitalization because they are markers of disease aggressiveness and severity. In a population-based study from Olmsted County, MN, USA, factors associated with time to first hospitalization included ileocolonic disease (hazard ratio [HR] 3.3; 95% CI 1.8–5.8), small bowel disease [HR 3.4; 95% CI 1.9–6.1] and gastroduodenal disease [HR 4.0; 95% CI 1.2–13.8] as opposed to colitis.^21^ Compared with non-penetrating and non-stricturing disease, penetrating disease also increased the risk of first major abdominal surgery [HR 2.7; 95% CI 1.1–6.7].^21^ Similar findings have been reported in other cohorts.^49,51,54^

### 3.4. Question 4: What are the prognostic factors for intestinal failure or permanent stoma in CD?

**Table:** 

**Summary statement**		**Agreement [score 7–9], *n*/*N* [%]**
1	CD involving the rectal, perianal and /or perineal regions, particularly stricturing and complex fistulizing disease, is a risk factor for permanent stoma [EL3].	62/71 [88%]

Permanent stoma may be a therapeutic necessity in patients with refractory rectal or perianal CD and severe Cohn’s proctocolitis. Identification of risk factors at an earlier stage of disease may guide more intensive medical intervention, preventing the development of strictures and fistulae that ultimately result in the need for irreversible surgery. In our review of the evidence [primarily in patients with perianal and/or colonic CD], the most frequent factors independently associated with a permanent stoma were: complex perianal fistulae,^55,56^ anal-canal stricture,^57^ perineum involvement and perineal granulomas,^58^ perianal sepsis,^59^ faecal incontinence,^55^ colonic CD^59^ and distal colonic involvement.^57,60^ Additionally, patients undergoing rectal resection or temporary faecal diversion for perianal disease control have a higher rate of permanent faecal diversion.^55,61^ Patients with permanent stoma have typically experienced a greater number of previous abdominal surgeries than those without a permanent stoma.^55,59^ It is important to acknowledge that the likelihood of successful stoma reversal after temporary diversion for control of perianal sepsis or disease is low.

Intestinal failure is a rare complication of CD, with a lack of consistent risk factors reported in the literature. Nevertheless, bowel-preserving strategies, such as strictureplasty or stricture balloon dilation, should be used whenever possible to prevent intestinal failure.

### 3.5. Question 5: What are the prognostic factors for proximal disease extension in UC?

**Table:** 

**Summary statement**		**Agreement [score 7–9], *n*/*N* [%]**
1	Clinical factors (delay in diagnosis of >6 months [EL3], family history of IBD [EL3], young age at diagnosis and disease severity), need for steroids at diagnosis, poor response to therapy [>3 relapses per year] and concurrent primary sclerosing cholangitis [PSC] may be associated with increased risk of proximal disease extension in UC [EL4].	53/70 [76%]

UC is a dynamic disease, with up to 50% of patients progressing from limited forms of disease [proctitis, left-sided colitis] to more extensive forms of colitis [extensive colitis, pancolitis].^62,63^ The extent of colitis is clinically relevant, as extensive colitis is associated with higher hospitalization rates, need for corticosteroids, greater likelihood of surgery, and increased risk of progression to dysplasia and colorectal cancer [CRC]. The ability to identify patients who are likely to experience disease extension would allow close monitoring and tight control, and perhaps more intensive treatment. It is important to note that most studies on this topic address association of potential risk factors with extensive colitis, rather than predicting proximal extension of disease, and do not allow assessment of whether the same factors that cause proximal disease extension are also predictive of extensive disease from the onset. This analysis was limited to studies that specifically evaluated risk factors for proximal disease extension [Table 2]. In a paediatric cohort with UC, a delay in diagnosis of more than 6 months and a family history of IBD were associated with increased risk of proximal disease extension [OR 5.0; 95% CI 1.2–21.5 and OR 11.8; 95% CI 1.3–111.3, respectively].^64^ In an adult cohort, independent factors associated with disease proximal extension were younger age at diagnosis [HR 0.98, 95% CI 0.96–0.999] and the presence of PSC [HR 12.83, 95% CI 1.36–121.10].^65^ More than three relapses in one year, a requirement for systemic steroid or immunosuppressive treatment and non-smoking were associated with risk of proximal extension in a retrospective cohort study of adults with UC.^66^ Increased severity of disease and use of corticosteroids upon diagnosis were significantly associated with proximal disease extension in adults with ulcerative proctitis.^67^

**Table 2.** Clinical, demographic and endoscopic prognostic predictors in ulcerative colitis [UC] and the associated impact on disease course.

**Prognostic factor**	**Impact**
Young age at diagnosis	More extensive disease [paediatric UC]^80 ^ Colectomy^64 ^ Proximal disease extension^64 ^ Acute severe UC^68^ Colorectal neoplasia^85 ^
Family history	Proximal disease extension [family history of IBD]^64 ^ Colorectal neoplasia [family history of CRC]^103 ^
Refractory proctitis [>3 relapses per year]	Proximal disease extension ^66 ^
Male sex	Colectomy^78 ^
Extensive colitis	Colectomy^68 ^ Acute severe UC^68 ^ Hospitalization ^82 ^ Colorectal neoplasia^89 ^
High histological inflammation score	Colorectal neoplasia^101 ^
Disease duration >10 years	Colorectal neoplasia^85,121 ^ Colectomy^24 ^
Steroid dependence/ resistance	Colectomy^69 ^ Hospitalization^122 ^
Smoking	Less need for hospitalization^72 ^ Proximal disease extension [protective]^66 ^ Protective from colectomy^73 ^
Concurrent infection [cytomegalovirus or *Clostridium difficile*]	Flare and hospitalization^76,77 ^
Primary sclerosing cholangitis	Colectomy^84 ^ Proximal disease extension^65 ^ Colorectal cancer^71 ^ Protective for hospitalization^70 ^

### 3.6. Question 6: What are the prognostic factors for acute severe UC?

**Table:** 

**Summary statements**		**Agreement [score 7–9], *n*/*N* [%]**
1	Extensive disease [EL2], younger age at diagnosis [EL3] and shorter duration of disease [EL2] are clinical risk factors for acute severe UC.	73/83 [88%]
2	PSC reduces the risk of hospitalization for UC flare [EL2].	34/44 [78%]
3	Active smokers have a reduced risk of hospitalization for UC flare [EL2].	71/86 [83%]
4	Extensive disease and concurrent infection with cytomegalovirus [EL3] or *Clostridium difficile* [EL4] are risk factors for hospitalization for UC flare.	76/85 [89%]

Acute severe UC is a medical emergency that requires intensive medical therapy or colectomy; however, robust prognostic factors for this event have not been established [Table 2]. In a retrospective analysis of a cohort of 750 patients in the UK diagnosed with UC from 1996 to 2001, acute severe UC occurred more frequently in patients with more extensive disease, a younger age at diagnosis and a shorter duration of disease.^68^ More recently, Cesarini *et al*. have shown that the likelihood of developing acute severe UC within 3 years of diagnosis was increased in patients with extensive colitis [E3 from Montreal classification] who presented with C-reactive protein [CRP] >10 mg/l and low haemoglobin [<13.5 g/dl for men or 12.1 g/dl for women].^69^ Data from this single-centre study require validation in external cohorts.

In the absence of studies evaluating acute severe UC as a definitive endpoint, hospitalization due to UC flares may act as a surrogate marker. A concurrent diagnosis of PSC may be a protective factor for hospitalization in UC patients,^70^ which is in accordance with studies suggesting that PSC carries a milder course of colonic activity in UC patients.^71^ Smoking has also been associated with a lower hospitalization rate in UC;^72^ conversely, quitting smoking increases the risk of hospitalization.^73,74^ Finally, extensive colitis,^75^ cytomegalovirus infection^76^ and *C. difficile* infection^77^ are risk factors for hospitalization for UC flare.

### 3.7. Question 7: What are the prognostic factors for colectomy in UC?

**Table:** 

**Summary statements**		**Agreement [score 7–9], *n*/*N* [%]**
1	Male sex [EL3] and early disease onset [EL3] are associated with colectomy in adults.	62/77 [81%]
2	Disease characteristics (extensive disease [EL3], disease of >10 years’ duration [EL3] and severe disease at index admission [EL3]), presence of PSC [EL2] and frequent hospitalization for severe UC flares [EL3] are clinical predictors for [all-cause] colectomy in UC.	72/82 [88%]
3	Active smoking reduces colectomy rates [EL2].	64/83 [77%]

In patients with UC, colectomy is undertaken in the emergency setting [typically for complications of fulminant colitis], in hospitalized patients who are non-responsive to maximal medical therapy and as elective treatment in patients who have persistent symptoms despite medical therapy, dysplasia or CRC. We recognize that it is important to distinguish prognostic factors according to colectomy context [Table 2].

In an analysis of the University of Manitoba IBD Epidemiology Database, predictors of early colectomy [≤90 days from diagnosis date] included male sex (HR 2.63; [corrected] 95% CI 1.58–4.36) and being initially diagnosed during hospitalization [HR 12.46; 95% CI 7.40–21.0].^78^ A review of the literature found that disease extent, particularly extensive disease^24,79^ and pancolitis,^80^ may predict subsequent colectomy. Longer disease duration was also identified as a risk factor, both in adults^24,81^ and in children.^80^ However, caution is needed when interpreting the evidence as the data are cumulative and are confounded by the differences in follow-up duration. Patients who are hospitalized at UC diagnosis^78,82^ or require recurrent hospitalization for UC management^82^ should be carefully monitored for colectomy indicators. While the presence of PSC reduces the risk of hospitalization for UC [discussed previously], it is also a risk factor for colectomy, primarily due to the associated risk of colorectal neoplasia.^83^ There is limited evidence that active smoking is protective against colectomy^84^ and smoking cessation in patients with established UC has not been shown conclusively to influence the rate of colectomy.^73^

### 3.8. Question 8: What are the prognostic factors for dysplasia and CRC in IBD?

**Table:** 

**Summary statements**		**Agreement [score 7–9], *n*/*N* [%]**
1	Duration of disease, extent of disease and PSC are associated with the development of CRC in colonic IBD [EL2].	80/84 [95%]
2	Persistent histological activity is associated with the development of dysplasia and CRC in UC [EL3].	76/81 [94%]
3	Family history of a first-degree relative with sporadic CRC is associated with the development of CRC in IBD [EL3].	72/84 [86%]
4	Male sex [EL2] is associated with the development of CRC in IBD; older age at diagnosis [EL3] is associated with a decreased time interval to CRC development in IBD.	61/78 [78%]

Early intervention with immunosuppressive or biological therapies may have contributed to an overall decline in the incidence of CRC in patients with IBD, although it is evident that some subgroups of patients with UC remain at increased risk of CRC.^85^ In patients with IBD, independent risk factors for the development of CRC or high-grade dysplasia include longer duration of IBD^85,86,87,88^ and greater extent of colonic involvement at diagnosis.^86,87,89^ The excess risk becomes significant 7–10 years after diagnosis in the general IBD population^90^ although it is immediately seen in patients with PSC.^85^ In UC, disease extent is also a predictor for CRC development, with increased risk in patients with pancolitis compared with left-sided or distal colitis.^87,89,91,92^ A number of studies have shown that the concurrent presence of PSC is a strong risk factor for dysplasia or CRC development,^85,93,94,95,96,97^ warranting special surveillance protocols. Furthermore, when assessing for site of cancer development, PSC was a predictor of cancer proximal to the splenic flexure.^96^ In a study in patients with colonic CD, the presence of PSC was weakly associated with the development of dysplasia or CRC.^98^

Two referral centre studies have demonstrated an association between histological inflammation score and the development of dysplasia or CRC in patients with UC,^99,100^ and one case-control study found that every 1-unit increase in histological score independently increased the odds of colorectal neoplasia by a factor of 4.69 [95% CI 2.10–10.48].^101^ The possible relationship between elevated CRP or erythrocyte sedimentation rate and CRC^102^ may also support the association between poorly controlled inflammation and cancer risk.

Among IBD patients with a first-degree relative with CRC, the relative risk [RR] of developing CRC was increased [RR 2.5; 95% CI 1.4–4.4] and remained elevated for both UC [RR 2.0; 95% CI 1.0–9.4] and CD [RR 3.7; 95% CI 1.4–9.4].^103^ Furthermore, the RR was higher for those with a first-degree relative diagnosed with CRC before age 50 [RR 9.2; 95% CI 3.8–23]. Male sex is independently associated with the development of CRC or high-grade dysplasia.^86,100,104^ While total disease duration is associated with the development of CRC, older age at diagnosis has been associated with shorter time to CRC onset.^86,89,93,105,106^

### 3.9. Question 9: What are the prognostic factors for death in IBD?

There are few identified predictor factors for mortality in IBD. A meta-analysis of 10 studies reported standardized mortality ratios [SMRs] in UC with a range from 0.7 to 1.4. The pooled ratio did not demonstrate an overall risk of dying that was different from the background population [pooled SMR 1.1; 95% CI 0.1–1.2].^107^ A similar meta-analysis of nine studies in CD reported a range of SMRs from 0.72 to 3.2, with a pooled SMR of 1.39 [95% CI 1.30–1.49] reflecting an increased overall mortality in patients with CD compared with the background population.^108^ The risk of death after adjustment for sex and smoking was compared in a large population cohort study including 16550 patients with IBD and 82917 matched controls; among patients with UC, those aged 40–59 years had the greatest increase in risk of death [HR 1.79; 95% CI 1.42–2.27], while in patients with CD, those aged 20–39 years had the largest statistically significant risk for death [HR 3.82; 95% CI 2.17–6.75].^109^ One cohort study found that age at diagnosis and male gender were independently associated with mortality in patients with UC; in patients with CD, only age at diagnosis was statistically significant.^110^ Another study found an independent association between PSC and mortality in IBD patients.^111^

## 4. Implications for practice

Stratifying patients and individualizing therapy in IBD should be an ongoing process. It is important for treating physicians and patients to understand which factors are associated with different outcomes as this may influence important therapeutic decisions. Evaluating these factors should arguably be the first step in stratifying patients into low-risk or high-risk groups and should drive treatment target discussions. It is equally important to actively pursue and document resolution of inflammation and adjust therapy accordingly [treat to target], offering the clinician the opportunity to improve patient outcomes at every stage of the disease process.^112^ Postponing adequate therapy in patients with aggressive disease may result in disease progression and complications; however, treating all patients intensively may lead to over-treatment and expose some patients to unnecessary risks of immunosuppression.^113^ Therefore, it is important to be aware of the risk factors associated with specific complications and to use these to tailor therapy. Briefly, patients with CD presenting with a young age at diagnosis, extensive anatomical involvement, deep ulcerations at endoscopy, ileal or ileocolonic involvement, perianal disease and/or severe rectal disease and penetrating or stenosing behaviour should be regarded as those with the highest probability of developing complications and therefore merit intensive therapy and close follow-up.^114^ Patients with UC presenting at young age at diagnosis, with extensive colitis and frequent flare-ups needing steroids or hospitalization bear a higher risk of colectomy. Concurrent presence of PSC, smoking status and superimposed infections may impact the course and activity of UC; therefore, these are clinical risk factors needing attention from the clinician.

## 5. Future directions

The use of prognostic factors to guide IBD management is an evolving field. We must be mindful that most of the currently available prognostic factors in IBD are clinical and lack precision. Furthermore, many of these risk factors have been identified retrospectively and have yet to be validated; appropriately powered prospective studies are still required to inform algorithms that can truly predict the risk for disease progression in the individual patient. While use of serological and genetic markers in prognostication has been hampered by lack of sensitivity and lack of wide availability, these are of increasing clinical interest. Indeed, composite scores incorporating clinical information and molecular profiling, and considering harder endpoints such as bowel damage^115,116^ and disability,^117^ will hopefully allow us to better personalize therapy in the future, both at diagnosis and throughout the disease course.^118^

## Funding

AbbVie provided funding to the Lucid Group, Loudwater, UK, to manage the IBD Ahead educational programme. AbbVie paid consultancy fees to the Global Steering Committee for their participation in the Global Steering Committee meeting in Copenhagen, Denmark, and speaker fees to the committee members who presented at the international IBD Ahead meeting in Frankfurt, Germany. Travel to and from these meetings was reimbursed. The bibliographical fellows were paid consultancy fees by AbbVie to conduct the literature reviews. No payments were made to the authors for the development or writing of this manuscript. Payment of fees for Open Access was made by AbbVie. Juliette Allport of the Lucid Group provided editorial support to the authors in the development of this manuscript; AbbVie paid the Lucid Group for this work.

## Conflict of Interest

JT: served as a speaker or consultant for AbbVie, Falk, Ferring, and Takeda. FC: served as a speaker or consultant for AbbVie, MSD and Takeda. KHK: served as a speaker for and received consultancy fees from AbbVie, MSD and Takeda. DM: none. TL: none. MZ: served as an advisory board member or speaker for AbbVie, Apsen, Janssen, Nestlé, Pfizer, Takeda and UCB Biopharma and received support for medical congresses from AbbVie, Janssen and Takeda. GVA: received consultancy and/or lecture fees from AbbVie, Aptalis, Bristol-Myers Squibb, Ferring, Janssen, MSD, Novartis, Takeda, UCB Pharma and Warner Chilcott. Research support to the University of Leuven has also been received from AbbVie, MSD and Zealand Pharma. JCL: served as a speaker or advisory board member for AbbVie, Falk and Ferring. JOL: served as a speaker, consultant or advisory board member for, or has received research funding from, AbbVie, Actavis [Warner Chilcott], Atlantic Healthcare, Ferring, Hospira, Janssen, MSD, Napp, Pfizer, Shire Pharmaceuticals, Takeda, Tillotts and Vifor Pharma. DTR: served as a consultant or received grant support from: AbbVie, Amgen, Genentech, Janssen, Pfizer, Prometheus Laboratories, Shire Pharmaceturicals, Takeda and UCB Pharma. RP: received consultant and/or lecture fees from AbbVie, Amgen, AstraZeneca, Axcan Pharma [now Aptalis], Biogen Idec, Bristol-Myers Squibb, Centocor, ChemoCentryx, Eisai Medical Research Inc., Elan Pharmaceuticals, Ferring, Genentech, GlaxoSmithKline, Janssen, MSD, Millennium Pharmaceuticals Inc. [now Takeda], Ocera Therapeutics Inc., Otsuka America Pharmaceutical, Pfizer, Shire Pharmaceuticals, Prometheus Laboratories, Schering-Plough, Synta Pharmaceuticals Corp, Teva, UCB Pharma and Warner Chilcott. JFC: served as consultant, advisory board member or speaker for AbbVie, ABScience, Amgen, Bristol-Myers, Squibb, Celgene, Celltrion, Danone, Dr. August Wolff, Ferring, Genentech, Giuliani SPA, Given Imaging, Immune Pharmaceuticals, Janssen, Kyowa Hakko Kirin Pharma, MedImmune, Merck & Co., Millennium Pharmaceuticals Inc., Navigant Consulting, Neovacs, Nestlé, Nutrition Science Partners, Pfizer, Prometheus Laboratories, Protagonist, Receptos, Sano, Schering Plough, Second Genome, Shire, Takeda, Teva Pharmaceuticals, TiGenix, UCB Pharma and Vertex.

## Author Contributions

This paper was developed from recommendations made by the IBD Ahead programme chairs [JFC and RP], literature review sub-committee [JFC, JCL, JOL, DR and GVA] and bibliographical fellows [FC, KK, TL, DM, JT and MZ]. AbbVie selected the programme chairs and reviewed the final draft of the manuscript. The authors maintained complete control over the content of the paper and it reflects their opinions. All authors made substantial contributions to [1] the conception and design of the study, or acquisition of data, or analysis and interpretation of data, [2] drafting the article or revising it critically for important intellectual content and [3] final approval of the version to be submitted.
